# Obesity and Multisite Pain in the Lower Limbs: Data from the Osteoarthritis Initiative

**DOI:** 10.1155/2020/6263505

**Published:** 2020-07-09

**Authors:** Vishal Vennu, Aqeel M. Alenazi, Tariq A. Abdulrahman, Ahmad S. Binnasser, Saad M. Bindawas

**Affiliations:** ^1^Department of Rehabilitation Sciences, College of Applied Medical Sciences, King Saud University, Riyadh, Saudi Arabia; ^2^Department of Physical Therapy and Rehabilitation Science, University of Kansas Medical Center, Lawrence, Kansas, USA; ^3^Department of Rehabilitation Sciences and Physical Therapy, Prince Sattam Bin Abdulaziz University, Alkharj, Saudi Arabia; ^4^Department of Orthopaedics, College of Medicine, King Saud University, Riyadh, Saudi Arabia

## Abstract

**Background:**

Although several studies investigated the relationship between obesity, osteoarthritis, and pain, no study examined the association between obesity and multijoint pain in the lower limbs. The purpose of this study was to address this gap.

**Method:**

This cross-sectional study was performed in Riyadh, Saudi Arabia, between March and April 2019. In this study, a total of 4,661 adults aged 45–79 years with or at high risk for knee osteoarthritis were included from the Osteoarthritis Initiative. The persons who had an elevated risk of developing symptoms of knee osteoarthritis during the study were defined as high risk for knee osteoarthritis. According to the body mass index, participants were categorized into three groups: normal weight (*n* = 1,068), overweight (*n* = 1,832), and obese (*n* = 1,761). Logistic regression was used to examine the association between obesity and multisite pain.

**Results:**

The odds of multisite pain was associated significantly (*p* < 0.001) by 1.36 times higher with obesity than normal weight, no, or sigle-site pain, even after adjusting for sociodemographic and health variables

**Conclusion:**

Obesity is associated with an increased likelihood of multisite pain in the lower limbs. The results enable clinicians to adopt better standards of practice for the prevention and screening of multisite pain in this community.

## 1. Introduction

The prevalence of obesity and its associated risks have risen substantially in several countries in recent decades [1, 2]. The World Health Organization (WHO) concluded that more than 1.5 billion adult men (39%) and women (40%) aged 18 years and older were overweight [3]. Of these, over 650 million (13%) of the world's adult population (11% men and 15% of women) were obese. A recently published report indicates a significant increase in obesity from 33.7% to 39.6% amongst adult Americans between the years 2007 and 2016 [4]. A recent study analysis has drawn attention to the fact that no countries have shown a significant decrease in obesity in the past 33 years [5]. This study suggests that approximately two in three of the world's obese people live in the developing world. The study also shows a high rate of obesity in Central America [5].

Obesity is associated with many comorbidities, especially for cardiovascular disease, cancer, diabetes, chronic kidney disease, and osteoarthritis (OA) [2, 6, 7]. However, a major global health problem of particular significance is musculoskeletal pain at multiple sites (multisite pain) [8]. Multisite pain is more prevalent than single-site pain and causes reduced quality of life and functional problems [2]. An increased risk of pain has been observed because of the rising epidemic of obesity [9]. The specific segment of the population that is affected is rising rapidly [9], especially in the United States (US) [10].

The main impact of obesity in people with OA is on the weight-bearing joints such as the hip, knee, ankle, or foot. However, a leading cause of disability in patients with OA worldwide is the primary symptom of the pain of that disease [11]. The previous scoping reviews have reported the impact of obesity on the progression and incidence of OA and cost-burden in the OA population [12, 13]. Also, these reviews described how weight loss prevents OA and OA-related symptoms, particularly pain.

Although earlier studies used different definitions of obesity and pain with various populations, much uncertainty still exists about the relationship between obesity and pain in the literature. For example, few epidemiological studies have confirmed the link between obesity and pain [14]. Previous meta-analyses reported the relationship between increased BMI and the risk of pain in the knee and hip [15]. Other factors such as age, sex, race, education, marital status, annual income, comorbidity, and depressive symptoms are associated with multisite pain [2] and also with obesity [8]. A survey showed a strong association between BMI and daily pain [14]. The association was found to persist even after controlling for age, gender, and the increased risk of pain with age. Another study found a significant association of an increase in knee pain with each level of obesity, even after adjusting for the severity of knee OA [16]. However, many studies have reported inconsistent associations between obesity and pain in different populations [2, 8, 17]. The US-based survey reported an increase in knee pain over two decades, independent of an individual's age and body mass index (BMI) [16].

To date, several studies have examined the association between obesity, OA-related pain [15, 16], and multisite pain in the general population [7, 8]. However, no previous study had investigated the association between obesity and multisite pain in the lower limbs among adults with or at high risk for knee OA [7, 14, 15]. Thus, the current study is intended to examine the association between obesity and multisite pain in the lower limbs, such as in the knee, hip, ankle, and foot among adults with or at high risk for knee OA.

## 2. Materials and Methods

In this cross-sectional study, a secondary analysis was performed using baseline data of 4,661 adults from the Osteoarthritis Initiative (OAI) database in compliance with the Declaration of Helsinki and appropriate reporting guidelines (see Figures S1 in the Supplementary Material for STROBE checklist for cross-sectional studies). The OAI is a multicenter initiative, as well as an ongoing prospective cohort study available for free public access. It is a public-private partnership between the National Institute of Health and the private industry sector. The OAI study aims to improve both the diagnosis and monitoring of knee OA and evaluating new treatments for this condition. Participants in the OAI were enrolled from 4 clinical sites across the US (Baltimore, MD; Pittsburgh, PA; Pawtucket, RI; and Columbus, OH) between February 2004 and May 2006. All potential participants who qualified at the phone interview were interviewed face-to-face at the clinical sites by trained staff. The Committee on Human Research of the Institutional Review Board (IRB) for the University of California, San Francisco (UCSF), and its affiliates approved the study protocol (approval number: FWA00000068). All participants were asked to read and sign the informed consent form before enrolment into the original OAI study.

In this study, we included males and females aged 45–79 years with or at high risk for knee OA, irrespective of race. The participants with knee OA were defined as symptomatic tibiofemoral knee OA at baseline. The participants with a high risk for knee OA were described as no symptomatic tibiofemoral OA in either knee at baseline. Still, they had an elevated risk of developing symptoms of knee OA during the study. Participants with no radiographic findings and no eligibility risk factors for OA, as well as those who were underweight (<18 kg/m^2^), or unable to provide the relevant data were excluded.

The data collected using Statistical Analysis Software (SAS) for analysis include sociodemographic information (e.g., age, gender, race, educational status, marital status, employment status, and annual income status) and health status (e.g., comorbidity and depressive symptoms). The Charlson comorbidity index was used for assessing and classifying the comorbidity into two levels (0 versus ≥1) [18]. The Center for Epidemiological Studies Depression (CESD) Scale score of 16 or higher was used to define the depressive symptoms [19]. The validity and reliability of these two data collections tools have been reported previously [1, 20].

Participants' weights were measured twice during clinic visits without the wearing of shoes or heavy jewelry or their being in possession of a wallet. Participants were also required to wear lightweight clothing. Height was measured twice in millimeters (mm) without shoes, using a stadiometer. The WHO criteria were used to classify participants into different weight categories [21]: normal weight (18.5–24.9; *n* = 1,068), overweight (25–29.9; *n* = 1,832), and obese (≥30 kg/m^2^; *n* = 1,761) [22].

Pain in the lower limbs was defined based on the answer “yes” in response to the following interviewer-administered question: “During the past 12 months, have you ever experienced any pain, aching, or stiffness either in or around your right/left knee?” The subject was then asked the same question to their right and left hip, ankle, and foot. A participant was recorded as having multisite pain if they answered “yes” to the above questions related to two or three out of four lower limbs. They were recorded as having only single-site pain in either knee, hip, ankle, or foot if they answered “yes” to the question relating to these joints. If participants answered “no” to the above questions, they were recorded as having no pain in any joint, such as the knee, hip, ankle, and foot. The participants were classified into one of two categories based on the presence or absence of pain in the lower limbs, such as the knee, hip, ankle, and foot. The first group, no or single-site pain (*n* = 2,946), was defined as no or single-site pain in any of the lower limbs. The second group, multisite pain (*n* = 1,716), was defined as pain in two or three out of four lower limbs. These classifications were based on previously published studies [7, 8]. The previous research has also shown the number of pain sites to be a helpful approach to classifying multisite pain and also showed that multisite pain varies in its uncertainty to pain at one location [23].

### 2.1. Statistical Analysis

All categorical data were expressed as frequencies, whereas all continuous data were expressed as means and standard deviations (SD). Either Pearson's chi-squared test, a post hoc test, or analysis of variance (ANOVA) test was used to compare variables between the groups. The distribution of frequency, percentage, and significance according to pain status in the lower limbs between normal weight, overweight, and obese was tested using the Cochran–Armitage test. The associations between obesity and multisite pain were assessed using logistic regression. The regression analyses included adjustments for age, sex, race, education, marital status, annual income, comorbidity, and depressive symptoms. The odds ratios (OR) and 95% confidence intervals (CI) were calculated for the overweight and obese groups. Participants in the normal weight and no or single-site pain groups served as the reference groups for the analyses. All analyses were completed utilizing SAS for Windows®, version 9.2 (SAS Institute, Inc., NC, USA). A *p*-value ≤0.05 is defined as statistically significant.

## 3. Results

**Figure 1:**
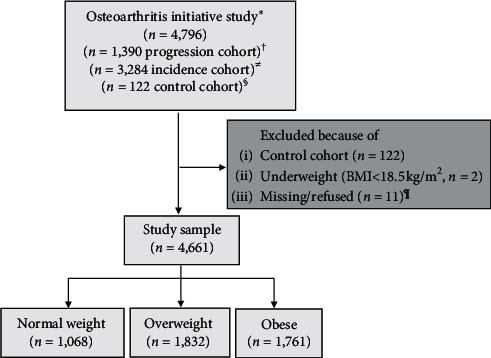
The flow of samples from the Osteoarthritis Initiative Study^*∗*^. ^†^Participants with symptomatic tibiofemoral knee osteoarthritis at baseline. ^≠^Participants with no symptomatic tibiofemoral osteoarthritis in either knee at baseline, but had an elevated risk of developing symptoms of knee osteoarthritis during the study. ^§^Participants with no pain, aching, or stiffness in either knee in the past year, along with no radiographic findings of osteoarthritis and no eligibility risk factors. ^¶^Data are missing for the variable indicating which knee was eligible for the study. BMI: body mass index.

Figure 1 illustrates the flow of the present study sample. Of the 4,796 recruited in the OAI, 4,661 participants were included in the current study. Of these, 1,068 (22.9%) had a healthy weight, 1,832 (39.3%) were deemed to be overweight, and 1,761 (37.8%) were obese.

The participants' baseline and clinical-demographic characteristics, according to their BMI, are presented in Table 1. Obese adults were significantly two years younger with an average age of 60 years than those with normal (61.8 years) and overweight (62.2 years) persons. Most of the adults in the obese group significantly had higher comorbidities (33.1%) and depressive symptoms (12.3%).

The participant's characteristics between those with and without multijoint pain are summarized in Table 2. The majority of females (67.3%) significantly had multisite pain. Persons with multisite pain had significantly higher BMI than those individuals with no or single-site pain. Most of the adults with multisite pain significantly had higher comorbidities (33.6%) and depressive symptoms (13.2%).

**Figure 2:**
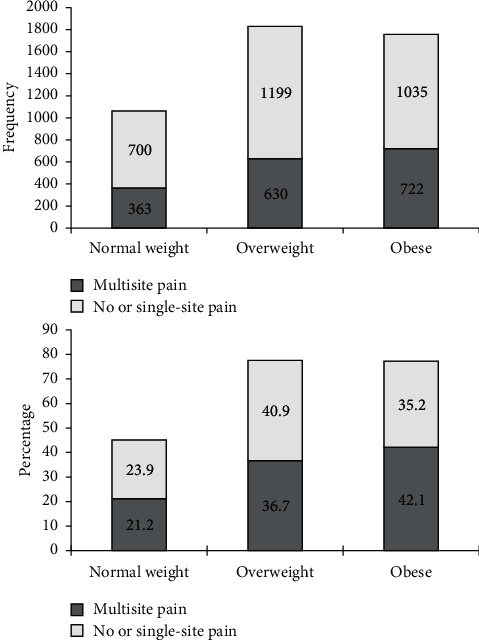
The distribution of frequency and percentages by pain status in the lower limbs between normal weight, overweight, and obese participants, *n* = 4,661. Cochran–Armitage test,*p* < 0.005.

The distribution and difference in multisite pain between normal weight, overweight, and obese participants were statistically significant (*p* < 0.0001). The majority of persons with obesity significantly had multisite pain (42.1%). Most of the persons who were overweight significantly had no or single-site pain (40.9%) (Figure 2).

In the unadjusted model, obesity was significantly associated with a 1.34-fold (95% CI = 1.14–1.58, *p* < 0.0001) increase in multisite pain compared with the normal weight group. After adjustment for age, sex, race, education, marital status, annual income, comorbidity, and depressive symptoms, there was also a significant association between obesity and multisite pain with a 1.36-fold (95% CI = 1.14–1.60, *p* 0.002) increase compared with the normal weight group (Table 3).

## 4. Discussion

The current study investigated the association between obesity and multisite pain in the lower limbs among adults with or at high risk for knee OA. The study findings demonstrated that obesity was significantly associated with an increased likelihood of multisite pain in the lower limbs compared to normal weight subjects. However, no significant association was found between being overweight and multisite pain in the lower limbs among this study group.

In this study, the finding may be due to mechanical overload and stress, as well as certain chemicals released from fat, all of which seem to contribute to inflammation in weight-bearing joints [25]. In support of our results, Bijlsma et al. provided evidence that obesity modulates joint pain through multiple components, including mechanical loading, inflammation, and mental status [9]. Incidentally, joint pain and dysfunction were found to be more significant at every successive level of obesity among patients with end-stage OA [26]. Moreover, the onset of obesity is reported to be a risk factor for joint pain and future disability due to poor physical fitness [27]. It is, therefore, reasonable to propose that maintaining a healthy weight may eliminate joint pain amongst adults.

The current study results are broadly consistent with the findings of earlier studies [28]. For example, a study of US adults (*n* = 430,912) revealed that different levels of obesity, particularly class III obesity, are more likely to be associated with joint pain and functional impairment [29]. Additionally, the study showed that, of the morbidly obese respondents, more than 60% suffered from joint pain. Furthermore, every successive level of obesity increased the odds of joint pain, particularly in Asians compared to Caucasians [30]. All of these studies examined the relationships between BMI and pain (either OA pain or another type of pain) in different populations. However, the findings are inconsistent with the study that showed an unclear association between being overweight or obese and future multisite pain in the general population [8].

The effects of obesity and mechanism of effect of obesity on the musculoskeletal system were well described previously [21]. Earlier studies also showed that obesity is likely to have a more substantial impact on musculoskeletal conditions, especially in women compared to men [24, 31–33]. Therefore, the current study findings may assist clinicians in adopting standards of practice for the management of obesity and pain in the lower limbs. Moreover, these standards could help individuals to achieve and maintain ideal body weight and avoid obesity-related complications, such as joint pain [30]. Scientific evidence has shown that obesity is a modifiable risk factor, and obese individuals have reported both a decrease in pain and a positive change in physical function due to weight loss combined with regular physical activity [34]. It is logical, therefore, to assume that maintaining a healthy BMI lowers the risk of joint pain.

The strengths of the study include the use of data from the OAI, which is an extensive, ongoing, public, and privately funded longitudinal research database. There are also limitations to the study. Firstly, the cross-sectional design is restricted to a single time point and may limit the generalizability of results to other regions. Secondly, we did not include waist circumference (WC) measurements in our analyses; these may have been a preferred measure of obesity for predicting disability [35]. Finally, because the multisite pain was self-reported by the participants, reporting bias may have occurred.

## 5. Conclusions

Obesity was the only factor associated with an increased likelihood of multisite pain in the knee, hip, ankle, and foot among adults with or at high risk for knee OA. These results may be of clinical significance in the treatment of obesity and joint pain in this population. Further longitudinal studies are needed, however, in order to understand the risk factors associated with obesity and multisite pain.

## Figures and Tables

**Table 1:** The demographic characteristics of the study participants according to their body mass index.

Characteristics	All (*N* = 4,661) Mean ± SD^*∗*^	Normal weight (*n* = 1,068)	Overweight (*n* = 1,832)	Obese (*n* = 1,761)	*p* ^*∗∗*^
Age (years)	61.3 ± 9.1	61.8 ± 9.5	62.2 ± 9.3	60.0 ± 8.6	<0.001
BMI (Kg/m^2^)	27.9 ± 2.0	22.8 ± 1.5	27.4 ± 1.4	33.7 ± 3.1	<0.001
	*n* (%)^*∗∗∗*^	*n *(%)^*∗∗∗*^	*n *(%)^*∗∗∗*^	*n *(%)^*∗∗∗*^	
Sex					<0.001
Male	1,942 (41.7)	338 (31.6)	888 (48.5)	716 (40.7)	
Female	2,719 (58.3)	730 (68.4)	944 (51.5)	1,045 (59.3)	
Race					<0.001
Caucasian	3.669 (78.7)	950 (89)	1,508 (82.3)	1,211 (68.8)	
African Americans/Asians/others	992 (21.3)	118 (11)	324 (17.7)	550 (31.2)	
Educational status					<0.001
Primary school or less	167 (3.6)	9 (1)	71 (3.9)	87 (4.9)	
High school or more	4,494 (96.4)	1,059 (99)	1,761 (96.1)	1,674 (95.1)	
Marital status					<0.001
Married	3,074 (66)	736 (68.8)	1,258 (68.7)	1,080 (61.3)	
Unmarried/divorced/widowed	1,587 (34)	332 (31.2)	574 (31.3)	681 (38.7)	
Employment status					0.026
Employed	2,883 (61.9)	639 (59.8)	1,112 (60.7)	1,132 (64.3)	
Not employed	1,778 (38.1)	429 (40.2)	720 (39.3)	629 (35.7)	
Income status					<0.001
≥$50,000/year	2,912 (62.5)	720 (67.4)	1,179 (64.4)	1,013 (57.5)	
<$50,000/year	1,749 (37.5)	348 (32.6)	653 (35.6)	748 (42.5)	
Charlson comorbidity index					<0.001
0	3,442 (73.8)	857 (80.2)	1,407 (76.8)	1,178 (66.7)	
≥1	1,219 (26.2)	211 (19.8)	425 (23.2)	583 (33.1)	
Depressive symptoms					0.001
CESD <16	4,188 (89.8)	971 (90.9)	1,672 (91.3)	1,545 (87.7)	
CESD ≥16	473 (10.2)	97 (9.1)	160 (8.7)	216 (12.3)	

^*∗*^Values are presented as means ± standard deviation (SD). ^*∗*^Pearson's chi-squared test, post hoc test, or analysis of variance (ANOVA) test was used to determine *p* values. ^*∗∗∗*^Values are presented as number (percent). BMI, body mass index; $, the United States dollar; CESD, Center for Epidemiological Studies Depression.

**Table 2:** The demographic characteristics of the study participants with the presence or absence of pain in the lower limbs.

Characteristics	All (*N* = 4,661) Mean ± SD^*∗*^	No or single-site pain (*n* = 2,946) Mean ± SD^*∗*^	Multisite pain (*n* = 1,716) Mean ± SD^*∗*^	*p* ^*∗∗*^
Age (years)	61.2 ± 9.1	61.4 ± 9.3	61.0 ± 8.9	0.087
BMI (kg/m^2^)	28.8 ± 4.8	28.4 ± 4.6	29.2 ± 5.0	<0.001
	*n* (%)^*∗∗∗*^	*n* (%)^*∗∗∗*^	*n* (%)^*∗∗∗*^	
Sex				<0.001
Male	1,944 (41.7)	1,383 (46.9)	561 (32.7)	
Female	2,718 (58.3)	1,563 (53.1)	1,155 (67.3)	
Race				0.143
Caucasian	3,667 (78.7)	2,337 (79.3)	1,330 (77.5)	
African Americans/Asians/other	995 (21.3)	609 (20.7)	386 (22.5)	
Educational status				0.366
Primary school or less	167 (3.6)	100 (3.4)	67 (3.9)	
High school or more	4,495 (96.4)	2,846 (96.6)	1,649 (96.1)	
Marital status				0.012
Married	3,071 (66)	1,980 (67.2)	1,091 (63.6)	
Unmarried/divorced/widowed	1,591 (34.1)	966 (32.8)	625 (36.4)	
Employment status				0.039
Employed	2,888 (61.9)	1,858 (63.1)	1,030 (40)	
Not employed	1,774 (38.1)	1,088 (36.9)	1,030 (60)	
Income status				0.002
≥$50,000/year	2,909 (62.4)	1,898 (64.4)	1,011 (58.1)	
<$50,000/year	1,753 (37.6)	1,048 (35.6)	705 (41.1)	
Charlson comorbidity index				<0.001
0	3,445 (73.9)	2,271 (77.1)	1,174 (68.4)	
≥1	1,217 (26)	675 (22.9)	542 (31.6)	
Depressive symptoms				<0.001
CESD <16	4,187 (89.8)	2,697 (91.5)	1,490 (86.8)	
CESD ≥16	475 (10.2)	249 (8.5)	226 (13.2)	

^*∗*^Values are presented as means ± standard deviation (SD). ^*∗∗*^Independent *t*-test was used to determine *p* values. ^*∗∗∗*^Values are presented as number (percent). BMI, body mass index; $, the United States dollar; CESD, Center for Epidemiological Studies Depression.

**Table 3:** The association between obesity and multisite pain in the lower limbs, *n* = 4,650^*∗*^.

BMI classification^†^	(*n*)	Unadjusted			Adjusted^≠^		
		OR	95% CI	*p*	OR	95% CI	*p*
Normal weight	(1,064)	1.00			1.00		
Overweight	(1,829)	1.01	0.86–1.19	0.338	1.12	0.95–1.32	0.586
Obese	(1,757)	1.34	1.14–1.58	<0.001	1.36	1.14–1.60	0.002

^*∗*^Data (*n* = 11) are missing from the variable indicating multijoint OA status. ^†^BMI = body mass index (kg/m^2^): normal weight (BMI = 18.5–24.9), overweight (BMI = 25–29.9), and obese (BMI ≥30.0). ^≠^Adjusted for age, sex, race, education, marital status, annual income, comorbidity, and depressive symptoms. OR, odds ratio; CI, confidence interval.

## Data Availability

The datasets generated and/or analysed during the current study are available in the National Institutes of Health repository (https://oai.nih.gov).

## Supplementary Materials

Supplementary Materials Figures S1: STROBE checklist for cross-sectional studies.

## References

[1] Katz J. N., Chang L. C., Sangha O., Fossel A. H., Bates D. W. (1996). Can comorbidity be measured by questionnaire rather than medical record review?. *Medical Care*.

[2] Kamaleri Y., Natvig B., Ihlebaek C. M., Benth J. S., Bruusgaard D. (2008). Number of pain sites is associated with demographic, lifestyle, and health-related factors in the general population. *European Journal of Pain*.

[3] World Health Organization (2016). Obesity and overweight factsheet from the who. https://www.who.int/news-room/fact-sheets/detail/obesity-and-overweight.

[4] Hales C. M., Fryar C. D., Carroll M. D., Freedman D. S., Ogden C. L. (2018). Trends in obesity and severe obesity prevalence in US youth and adults by sex and age, 2007-2008 to 2015-2016. *JAMA*.

[5] Ng M., Fleming T., Robinson M. (2014). Global, regional, and national prevalence of overweight and obesity in children and adults during 1980–2013: a systematic analysis for the global burden of disease study 2013. *Lancet*.

[6] Veronese N., Stubbs B., Solmi M. (2017). Association between lower limb osteoarthritis and incidence of depressive symptoms: data from the osteoarthritis initiative. *Age and Ageing*.

[7] Brady S. R. E., Mamuaya B. B., Cicuttini F. (2015). Body composition is associated with multisite lower body musculoskeletal pain in a community-based study. *The Journal of Pain*.

[8] Magnusson K., Østerås N., Mowinckel P., Natvig B., Hagen K. B. (2014). No strong temporal relationship between obesity and multisite pain - results from a population-based 20-year follow-up study. *European Journal of Pain*.

[9] Bijlsma J. W., Berenbaum F., Lafeber F. P. (2011). Osteoarthritis: an update with relevance for clinical practice. *The Lancet*.

[10] Nguyen D. T., Zhang Y., Zhu Y., Niu J., Zhang B., Felson D. T. (2011). Increasing prevalence of knee pain and symptomatic knee osteoarthritis: survey and cohort data. *Annals of Internal Medicine*.

[11] Neogi T. (2013). The epidemiology and impact of pain in osteoarthritis. *Osteoarthritis and Cartilage*.

[12] Bliddal H., Leeds A. R., Christensen R. (2014). Osteoarthritis, obesity and weight loss: evidence, hypotheses and horizons-a scoping review. *Obesity Reviews*.

[13] Flego A., Dowsey M. M., Choong P. F., Moodie M. (2016). Addressing obesity in the management of knee and hip osteoarthritis - weighing in from an economic perspective. *BMC Musculoskelet Disord*.

[14] Stone A. A., Broderick J. E. (2012). Obesity and pain are associated in the United States. *Obesity*.

[15] Abbate L. M., Stevens J., Schwartz T. A., Renner J. B., Helmick C. G., Jordan J. M. (2006). Anthropometric measures, body composition, body fat distribution, and knee osteoarthritis in women. *Obesity*.

[16] Weiss E. (2014). Knee osteoarthritis, body mass index and pain: data from the Osteoarthritis Initiative. *Rheumatology*.

[17] Hitt H. C., McMillen R. C., Thornton-Neaves T., Koch K., Cosby A. G. (2007). Comorbidity of obesity and pain in a general population: results from the Southern Pain Prevalence Study. *The Journal of Pain*.

[18] Jiang L., Rong J., Wang Y. (2011). The relationship between body mass index and hip osteoarthritis: a systematic review and meta-analysis. *Joint Bone Spine*.

[19] Jiang L., Tian W., Wang Y. (2012). Body mass index and susceptibility to knee osteoarthritis: a systematic review and meta-analysis. *Joint Bone Spine*.

[20] Blalock S. J., DeVellis R. F., Brown G. K., Wallston K. A. (1989). Validity of the center for epidemiological studies depression scale in arthritis populations. *Arthritis & Rheumatism*.

[21] Anandacoomarasamy A., Fransen M., March L. (2009). Obesity and the musculoskeletal system. *Current Opinion in Rheumatology*.

[22] Deshpande B. R., Katz J. N., Solomon D. H. (2016). Number of persons with symptomatic knee osteoarthritis in the US: impact of race and ethnicity, age, sex, and obesity. *Arthritis Care & Research*.

[23] Coggon D., Ntani G., Palmer K. T. (2013). Patterns of multisite pain and associations with risk factors. *Pain*.

[24] De Angelis G., Chen Y. (2013). Obesity among women may increase the risk of arthritis: observations from the Canadian Community Health Survey, 2007-2008. *Rheumatology International*.

[25] Nuttall F. Q. (2015). Body mass index. *Nutrition Today*.

[26] Sach T. H., Barton G. R., Doherty M., Muir K. R., Jenkinson C., Avery A. J. (2007). The relationship between body mass index and health-related quality of life: comparing the EQ-5D, EuroQol VAS and SF-6D. *International Journal of Obesity*.

[27] Nelson A. E., Renner J. B., Golightly Y. M. (2012). Differences in multi-joint symptomatic osteoarthritis phenotypes by race and gender: the Johnston County osteoarthritis project. *Osteoarthritis and Cartilage*.

[28] Swinburn B. A., Sacks G., Hall K. D. (2011). The global obesity pandemic: shaped by global drivers and local environments. *The Lancet*.

[29] Sun J., Zhou W., Gu T., Zhu D., Bi Y. (2018). A retrospective study on association between obesity and cardiovascular risk diseases with aging in Chinese adults. *Science Report*.

[30] Yang H. K., Han K., Kwon H. S. (2016). Obesity, metabolic health, and mortality in adults: a nationwide population-based study in Korea. *Science Report*.

[31] Hart H. F., van Middelkoop M., Stefanik J. J., Crossley K. M., Bierma-Zeinstra S. (2020). Obesity is related to incidence of patellofemoral osteoarthritis: the cohort hip and cohort knee (check) study. *Rheumatology International*.

[32] Müller R., Kull M., Põlluste K. (2017). The metabolic profile in early rheumatoid arthritis: a high prevalence of metabolic obesity. *Rheumatology International*.

[33] Hozumi J., Sumitani M., Matsubayashi Y. (2016). Relationship between neuropathic pain and obesity. *Pain Research and Management*.

[34] Haslam D. W., James W. P. (2005). Obesity. *Lancet*.

[35] Afshin A., Forouzanfar M. H., Reitsma M. B. (2017). Health effects of overweight and obesity in 195 countries over 25 years. *The New England Journal of Medicine*.

